# Ubiquitination of HTLV-I Tax in response to DNA damage regulates nuclear complex formation and nuclear export

**DOI:** 10.1186/1742-4690-4-95

**Published:** 2007-12-14

**Authors:** Michael L Gatza, Tajhal Dayaram, Susan J Marriott

**Affiliations:** 1 Department of Molecular Virology and Microbiology, Interdepartmental Program in Cell and Molecular Biology, Baylor College of Medicine, Houston, Texas 77030, USA

## Abstract

**Background:**

The HTLV-I oncoprotein, Tax, is a pleiotropic protein whose activity is partially regulated by its ability to interact with, and perturb the functions of, numerous cellular proteins. Tax is predominantly a nuclear protein that localizes to nuclear foci known as Tax Speckled Structures (TSS). We recently reported that the localization of Tax and its interactions with cellular proteins are altered in response to various forms of genotoxic and cellular stress. The level of cytoplasmic Tax increases in response to stress and this relocalization depends upon the interaction of Tax with CRM1. Cellular pathways and signals that regulate the subcellular localization of Tax remain to be determined. However, post-translational modifications including sumoylation and ubiquitination are known to influence the subcellular localization of Tax and its interactions with cellular proteins. The sumoylated form of Tax exists predominantly in the nucleus while ubiquitinated Tax exists predominantly in the cytoplasm. Therefore, we hypothesized that post-translational modifications of Tax that occur in response to DNA damage regulate the localization of Tax and its interactions with cellular proteins.

**Results:**

We found a significant increase in mono-ubiquitination of Tax in response to UV irradiation. Mutation of specific lysine residues (K280 and K284) within Tax inhibited DNA damage-induced ubiquitination. In contrast to wild-type Tax, which undergoes transient nucleocytoplasmic shuttling in response to DNA damage, the K280 and K284 mutants were retained in nuclear foci following UV irradiation and remained co-localized with the cellular TSS protein, sc35.

**Conclusion:**

This study demonstrates that the localization of Tax, and its interactions with cellular proteins, are dynamic following DNA damage and depend on the post-translational modification status of Tax. Specifically, DNA damage induces the ubiquitination of Tax at K280 and K284. Ubiquitination of these residues facilitates the dissociation of Tax from sc35-containing nuclear foci, and stimulates nuclear export of Tax through the CRM1 pathway.

## Introduction

Human T-cell leukemia virus type I (HTLV-I) is the etiological agent of adult T-cell leukemia (ATL) [1,2]. Although the mechanisms that regulate HTLV-I-mediated cellular transformation have not been fully elucidated, it is clear that the viral oncoprotein Tax is a central component in this process [3]. Tax is a pleiotropic protein that can deregulate various cellular processes including gene expression, cell cycle progression, and DNA repair [3-5]. The ability of Tax to perturb these processes depends upon its ability to interact with and dysregulate the activities of numerous cellular proteins [6-10]. Recent evidence has indicated that these interactions are not static and, in fact, are influenced by cellular conditions that affect post-translational modification [11-14].

Tax is ubiquitously expressed in heterogeneous nuclear foci known as Tax Speckled Structures (TSS), as well as more diffusely in the cytoplasm [9,11,15]. Both the nuclear and cytoplasmic activities of Tax are essential for cellular transformation [4,9,16]. Recent reports have indicated that the recruitment and/or retention of Tax binding partners within TSS, and elsewhere in the cell, is regulated by the post-translational modification status of Tax [13,17,18]. When fused to a SUMO monomer, Tax is localized predominantly to TSS and sumoylation of Tax is required for the formation of nuclear foci containing both RelA/p300 and Tax [12,13]. Conversely, ubiquitination, or fusion of Tax to a ubiquitin monomer, targets Tax to the cytoplasm and is required for Tax binding to the IkappaB kinase complex and nuclear translocation of RelA [12,13]. Further, ubiquitination and sumoylation of Tax occurs on the same C-terminal lysine residues indicating that these modifications are mutually exclusive. Thus, the subcellular localization of Tax, as well as its ability to interact with specific cellular proteins, appear to be regulated by these post-translational modifications [12,13]. Despite these findings, it is unclear how the post-translational modification status of Tax is regulated.

We recently reported that the subcellular localization of Tax, as well as its ability to interact with specific cellular proteins, are dynamic and are susceptible to various genotoxic stress-inducing agents [11]. In response to stress, the number and intensity of TSS is reduced and Tax relocalizes to the cytoplasm. The subcellular localization of Tax following stress phenotypically resembles that of ubiquitinated Tax [12,13]. Therefore, we hypothesized that DNA damage induces ubiquitination of Tax, which subsequently regulates its subcellular distribution. Here we demonstrate that DNA damage results in increased mono-ubiquitination of Tax. Mutation of Tax amino acids known to be targets of ubiquitination (K280 and K284) inhibited DNA damage-induced ubiquitination of Tax, resulting in retention of Tax within nuclear foci that contain sc35, a splicing factor that was previously shown to be a component of TSS [9,11,15]. We also showed that cytoplasmic transport of ubiquitin-Tax (UB-Tax) fusion proteins is regulated through a CRM1-dependent mechanism [19-21]. Inhibition of the CRM1 nuclear export pathway resulted in an accumulation of UB-Tax in nuclear speckles. These results are consistent with previous findings showing that the redistribution of Tax in response to DNA damage is regulated by the CRM1 nuclear export pathway [11]. Collectively, these results demonstrate that the subcellular localization of Tax, and its interactions with cellular proteins, which respond dynamically to DNA damage [11], are regulated, in part, by ubiquitination of Tax at lysine residues 280 and 284.

## Results

### Cytoplasmic localization of ubiquitinated Tax is leptomycin B sensitive

**Figure 1:**
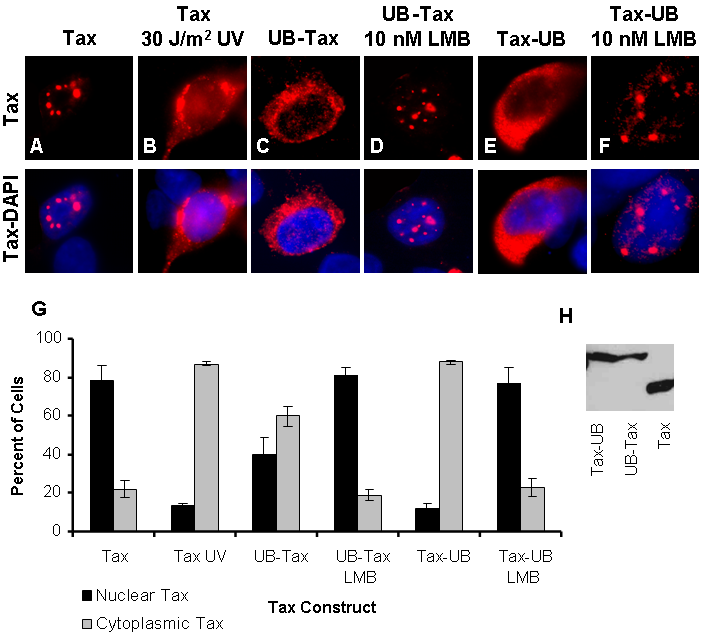
**Effect of ubiquitination on subcellular localization of Tax**. Tax localization alone (A-F, top row) or together with DAPI to visualize the nucleus (A-F, bottom row) was examined by immunofluorescent microscopy in transfected 293 cells. Tax is expressed in nuclear foci in the absence of DNA damage (A) and is localized in the cytoplasm following UV irradiation (B). The addition of a UB tag at either the N- (C) or C- (E) termini caused Tax to be localized in the cytoplasm but treatment with LMB (D and F) sequestered UB-Tax and Tax-UB to nuclear foci. All images are shown at a magnification of 63×. The percentage of cells expressing nuclear foci (black bar) or cytoplasmic Tax (gray bar) was scored in five hundred transfected cells, in three independent experiments (G). Western blot showing expression of UB-Tax, Tax-UB, and native Tax proteins (H).

The wild-type HTLV-1 Tax protein localizes predominately in nuclear foci known as Tax speckled structures (TSS). Previous studies have demonstrated that ubiquitination of Tax changes its distribution to a more diffuse, predominately cytoplasmic localization [12-14]. Since UV irradiation also causes a transient relocalization of Tax to the cytoplasm [11], we investigated the possibility that UV irradiation induces Tax ubiquitination. Consistent with previous reports, we found that Tax expressed in 293 cells formed nuclear foci under normal cell culture conditions (Figure 1A). Following UV irradiation (30 J/m^2^, 30 min) a rapid relocalization of Tax to the cytoplasm was observed, coincident with a decreased number and intensity of nuclear foci (Figure 1B). Ubiquitin-tags fused to the N- or C-terminus of Tax also localized to the cytoplasm of 293 cells (Figure 1C and 1E, respectively), although linkage of the Ub moiety to the C-terminus of Tax showed more cytoplasmic localization than did linkage to the N-terminus. The degree of cytoplasmic localization of these constructs could be due to tertiary structural effects of the Ub tag, or to differential effects of the Ub tag on protein interactions.

Tax contains both nuclear localization (NLS) and nuclear export (NES) sequences that enable it to shuttle between the nucleus and cytoplasm [11,22-24]. The cytoplasmic distribution of ubiquitin-Tax fusion proteins suggests that the ubiquitin modification either facilitates the export of Tax to the cytoplasm or that it inhibits the import of Tax to the nucleus. Either of these activities would result in the cytoplasmic accumulation of Tax. To distinguish between these possibilities, 293 cells expressing UB-Tax or Tax-UB were treated with leptomycin B (LMB), a CRM1 inhibitor [25,26] that has been shown to inhibit the export of Tax from the nucleus in response to UV irradiation [11]. The expression of UB-Tax, Tax-UB and native Tax is shown in Figure 1H. Tax localization was monitored by immunofluorescent microscopy. Following LMB treatment, both UB-Tax, (Figure 1D) and Tax-UB (Figure 1F) were observed predominantly in nuclear foci, similar to the distribution of wild-type Tax in unirradiated 293 cells (Figure 1A). The subcellular distribution of Tax in each panel was quantified by scoring five hundred transfected cells, from three independent experiments, for the presence of TSS and cytoplasmic Tax (Figure 1G). We, and others, have previously shown that LMB does not affect the subcellular localization of wild-type Tax [11,22]under normal cellular conditions. Taken together, these results suggest that Ub-Tax is exported using the CRM-1 pathway.

### UV irradiation increases the mono-ubiquitination of Tax

**Figure 2:**
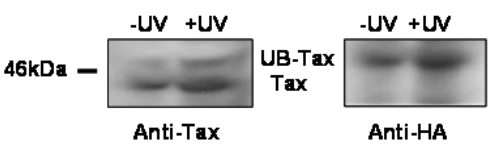
**UV irradiation increases Tax mono-ubiquitination**. Tax was immunoprecipitated from 293 cell lysates that had been transfected with Tax and HA-UB and either mock or UV irradiated (30 J/m^2^, 30 minute recovery). The immunoprecipitates were analyzed by immunoblot for Tax (left) or HA (right).

The finding that nuclear export of ubiquitinated Tax utilizes the CRM1 pathway (Figure 1), combined with our previous knowledge that nuclear export of Tax in response to DNA damage is mediated through a CRM1 dependent mechanism [11], suggested that Tax becomes ubiquitinated following DNA damage. To test this possibility, wild-type Tax and HA-tagged UB were transiently expressed in 293 cells for 48 hours and the cells were mock or UV (30 J/m^2^) irradiated. The ubiquitination status of Tax was examined by immunoprecipitating Tax from cellular lysates thirty minutes after irradiation. Lysates were analyzed by immunoblotting with α-Tax or α-HA antibodies to detect Tax and UB-Tax, respectively (Figure 2). Analysis of both unirradiated and UV-irradiated samples by anti-Tax immunoblot showed the expected 40 kDa Tax band as well as a slightly slower migrating band, with an approximate molecular mass of 48 kDa (Figure 2, left panel). The slower migrating band was also detected when the blot was re-probed with an anti-HA antibody (Figure 2, right panel). The slower migrating band is of the predicted molecular mass for monoubiquitinated Tax and was detected with both anti-Tax and anti-HA antibodies. Therefore, we conclude that this band represents mono-ubiquitinated Tax. Although ubiquitinated Tax was present in both irradiated and unirradiated cells, significantly more mono-ubiquitinated Tax was observed following UV irradiation (20.9% ± 2.1%) than in the absence of UV (p = 0.0033 paired t test). This was determined by calculating the ratio of mono-ubiquitinated (upper Tax band) to total Tax (upper and lower Tax bands) for both unirradiated and UV irradiated cells. These results demonstrate that mono-ubiquitination of Tax occurs following DNA damage and that this modification may regulate the relocalization of Tax following DNA damage.

### Lysine residues 280 and 284 regulate UV-induced Tax localization

**Figure 3:**
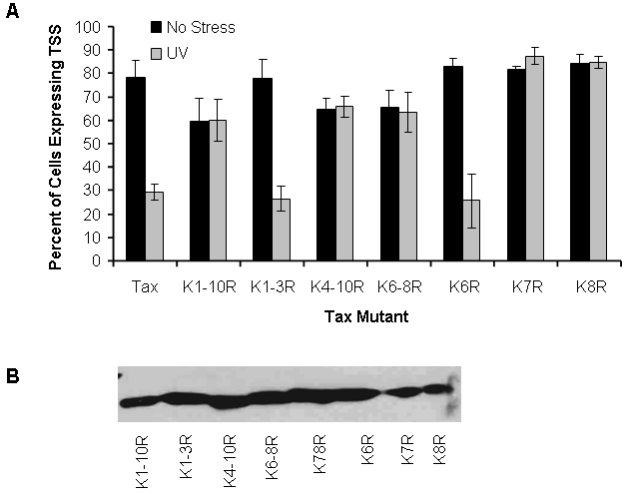
**Lysine residues 280 and 284 regulate UV-induced Tax localization**. (A) Wild-type Tax or Tax mutants were transiently expressed in 293 cells. Localization of Tax mutants in the absence (black bars) or presence of 30 J/m^2 ^UV irradiation (gray bars) is shown. Error bars represent the standard error in three independent experiments. (B) Western blot showing expression of each Tax mutant analyzed.

Ubiquitination of proteins occurs when an isopeptide bond is formed between a lysine residue on the target protein and ubiquitin [27]. Tax contains ten lysine residues at amino acids 85, 88, 111, 189, 197, 263, 280, 284, 324, and 346, which are referred to as lysine residues 1 through 10, respectively [12,28]. Since the increase in Tax ubiquitination following DNA damage correlated with redistribution of Tax to the cytoplasm, we predicted that lysine residues required for Tax ubiquitination, would also be required for translocation of Tax to the cytoplasm following DNA damage. To examine this possibility, the distribution of wild-type Tax and a Tax mutant containing arginine substitutions in all lysine residues (TaxK1-10R) was examined by immunofluorescent microscopy in mock or UV irradiated 293 cells (Figure 3). Consistent with our previous findings, the number of Tax-expressing cells containing nuclear Tax foci was significantly less in the presence (29.2% ± 3.4%) than in absence (78.1% ± 7.7%) of UV irradiation. However, the percentage of cells expressing nuclear foci in the presence (60.0% ± 8.9%) or absence (59.3% ± 10.2%) of UV irradiation was similar in cells expressing TaxK1-10R, in which all lysine residues were mutated. These results suggest that one or more of the lysine residues in Tax are required for Tax export from the nucleus following DNA damage.

To identify specific lysine residues that regulate nuclear export, the subcellular localization of a series of Tax mutants (Figure 3) was examined by immunofluorescent microscopy in mock and UV irradiated 293 cells. In cells expressing the TaxK4-10R mutant, which is resistant to ubiquitination, a similar percentage of nuclear foci were observed before (64.8% ± 4.7%) and after (65.9% ± 4.5%) UV irradiation. This result demonstrates that the TaxK4-10R mutant was not efficiently exported to the cytoplasm following UV irradiation. Thus, lysine residues in the C-terminus of Tax are the target of UV-induced ubiquitination and regulate nuclear export. Conversely, in cells expressing the TaxK1-3R mutant, the percentage of cells containing nuclear Tax declined from 77.6% ± 8.5% before irradiation to 26.4% ± 5.2% after irradiation. The observation that lysine residues in the amino-terminus of Tax are not required to regulate nuclear export is consistent with previous studies indicating that these residues are not required for Tax ubiquitination. Together, these results demonstrate that lysine residues within the C-terminus must be properly ubiquitinated in order for Tax to be exported from the nucleus following DNA damage [12,28].

The mutant, TaxK6-8R, was used to further map lysine residues within the carboxyl terminus of Tax that are required for DNA damage-induced nuclear export. Since the percentage of nuclear foci in cells expressing TaxK6-8R was similar in mock (65.5% ± 7.5%) and UV irradiated (63.4% ± 8.5) cells, one or more of lysine residues 263, 280, and/or 284 (K6, K7, K8) are required for Tax export in response to DNA damage. Point mutations within these individual residues, TaxK6R (K263R), TaxK7R (K280R), and TaxK8R (K284R), were used to define the role of individual lysine residues in nuclear export of Tax following DNA damage. The nuclear localization of TaxK6R resembled that of wild-type Tax before (83.4% ± 3.3%) and after (25.6% ± 11.6 %) UV irradiation. The efficient export of this mutant to the cytoplasm following UV irradiation suggests that it does not influence Tax localization in response to DNA damage. In contrast, the percentage of cells expressing TaxK7R that displayed predominantly nuclear Tax was similar before (81.8% ± 1.3%) and after (87.2% ± 3.7%) UV irradiation. The percentage of TaxK8R-expressing cells that demonstrated nuclear foci was also similar before (84.2% ± 3.9%) and after (84.7% ± 2.4%) UV irradiation. The failure of TaxK7R and TaxK8R mutants to change localization in response to UV irradiation suggests that lysine residues 280 (K7) and 284 (K8) play integral roles in the export of Tax from the nucleus following UV irradiation. Since these residues are known sites of ubiquitination, their failure to undergo nucleocytoplasmic shuttling in response to DNA damage may be a consequence of their inability to be ubiquitinated.

### Lysine residues 280 and 284 are ubiquitinated in response to DNA damage

**Figure 4:**
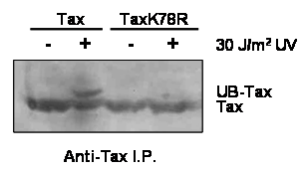
**Lysine residues 280 and 284 are required for UV-induced Tax ubiquitination**. Tax was immunoprecipitated from 293 cell lysates that had been transfected with Tax or TaxK7/8R, together with HA-UB. Cells were either mock (-) or UV irradiated (+) and the ubiquitination status of Tax was examined by immunoblot analysis for Tax.

Since Tax is ubiquitinated in response to DNA damage (Figure 2) and its subsequent translocation to the cytoplasm following UV irradiation depends on lysine residues 280 (K7) and 284 (K8) (Figure 3), we hypothesized that these residues are ubiquitinated in response to DNA damage. To determine whether mutation of K280 (K7) and K284 (K8) affected the ubiquitination of Tax following UV irradiation, plasmids encoding wild-type Tax or TaxK7/8R were transiently transfected, along with HA-tagged UB, into 293 cells and then mock or UV irradiated 48 hours after transfection. Lysates were prepared 30 minutes after irradiation, Tax was immunoprecipitated, and levels of Tax and UB-Tax and were examined by immunoblot using α-Tax (Figure 4) and α-HA (data not shown). As expected, following UV irradiation an increase in ubiquitinated Tax was observed in Tax expressing cells but not in cells expressing the TaxK7/8R mutant (Figure 4). These results demonstrate that lysine residues 280 and 284 are required for ubiquitination of Tax in response to DNA damage and further implicate these residues as being essential for nuclear export of Tax.

### Lysine residues 280 and 284 are required for the UV-induced dissociation of Tax from TSS

The sequestration of Tax in nuclear foci is thought to be mediated primarily through protein:protein interactions that depend on post-translational modifications of Tax [11,22]. We have demonstrated that mutations in C-terminal lysine residues prevent nuclear export of Tax and others have speculated that changes in the C-terminal conformation of Tax, as a consequence of either altered protein interactions or changes in post-translational modification status, are necessary for the Tax NES to be exposed [22]. Therefore, we examined whether mutation of specific residues within the C-terminus of Tax would affect nuclear export by altering the ability of cellular proteins to interact with Tax. To address this question, we used a previously characterized Tax mutant (M33), containing mutations at aa199-200 (S199A, L200S) within the nuclear export domain [24]. Both LMB treatment of wt Tax and mutation of the NES (TaxM33) prevented the nuclear export of Tax following DNA damage [11]. In addition, LMB treatment of wt Tax and mutation of the NES (TaxM33) reduced the colocalization of Tax with the TSS marker, sc35, in response to DNA damage. Therefore, we hypothesized that nuclear export of Tax is a multi-step process. In this model, Tax dissociates from TSS proteins prior to, and independent of, its interaction with CRM1 and the dissociation of Tax from TSS is stimulated by a stress-induced cellular signal. The Tax K280R (TaxK7R) and K284R (TaxK8R) mutants did not translocate to the cytoplasm in response to DNA damage despite the presence of an intact NES, suggesting that these residues (K280 and K284) and their post-translational modification may regulate the dissociation of Tax from TSS in response to DNA damage.

**Figure 5:**
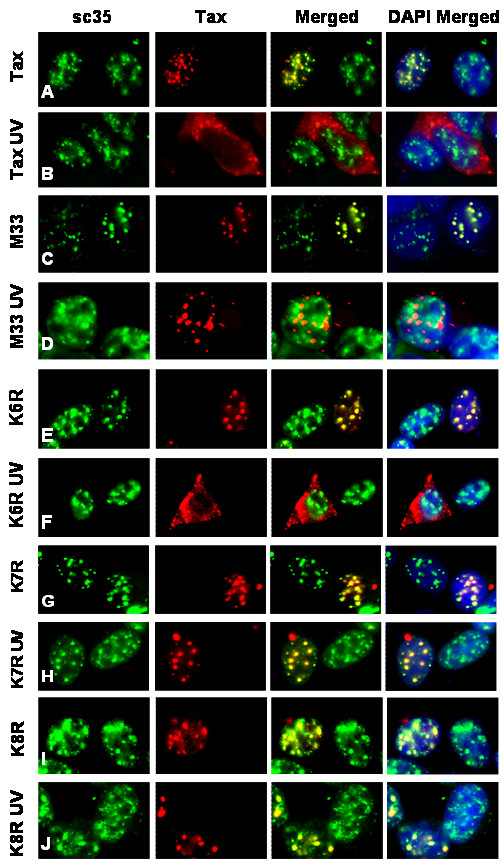
**Mutation of lysine residues 280 and 284 prevents UV-induced dissociation of Tax from TSS**. Wild-type (A and B), M33 (C and D), K6R (E and F), K7R (G and H), or K8R (I and J) Tax was transiently expressed in 293 cells for 48 hrs and subjected to 30 J/m^2 ^UV irradiation (B, D, F, H, J) or mock irradiated (A, C, E, G, I) and allowed to recover for 30 min. sc35 (left column) and Tax (left center column) were visualized by immunofluorescent microscopy separately, together (Merged, right center column), or together with DAPI (DAPI Merged, right column). All images are shown at a magnification of 63×.

**Figure 6:**
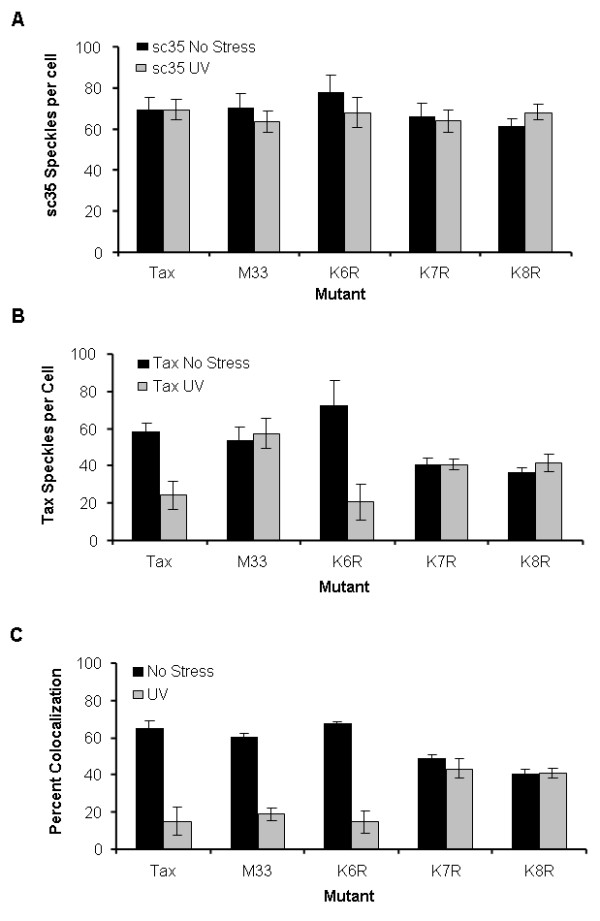
**Quantitation of Tax and sc35 nuclear speckles**. Nuclear foci (shown in Figure 5) containing sc35 (A), Tax (B), or colocalization (C) were quantitated in the absence (black bars) or presence of 30 J/m^2 ^UV irradiation (gray bars). Error bars represent the standard error in three independent experiments.

To determine whether the ubiquitination of Tax plays an integral role in its dissociation from TSS complexes following DNA damage, the colocalization of Tax mutants with sc35 was examined by immunofluorescent microscopy. Wild-type Tax, TaxM33, TaxK6R, TaxK7R, and TaxK8R were transiently expressed in 293 cells, the cells were mock or UV irradiated, and the localization of Tax and sc35 was analyzed by immunofluorescent microscopy after a 30 min recovery (Figure 5). Consistent with previous studies [11], wild-type Tax was extensively colocalized with sc35 under normal cellular conditions (Figure 5A and 6C). Following DNA damage, increased cytoplasmic localization and decreased colocalization of wild-type Tax with sc35 was observed (Figure 5B and 6C). Similarly, TaxK6R, which can be ubiquitinated, localized predominately in nuclear foci under normal cellular conditions (Figure 5E and 6B) where it was colocalized with sc35 (Figure 5E and 6C). As with wild-type Tax, following DNA damage, increased cytoplasmic localization of TaxK6R (Figure 5F and 6B) and reduced colocalization with sc35 (Figure 5F and 6C) was observed. In the absence of DNA damage, the Tax M33 mutant, which has a defective NES, was predominantly localized in nuclear foci containing sc35 (Figure 5C and 6B). In contrast to wild-type Tax and TaxK6R, the M33 mutant remained localized in nuclear foci even after DNA damage (Figure 5D and 6B). However, the M33 foci differed from TSS because they showed reduced co-localization with sc35 (Figure 5D). Interestingly, the TaxK7R and TaxK8R mutants were also present in nuclear foci following DNA damage but, in contrast to the M33 mutant, remained colocalized with sc35 (compare Figure 5G with 5H and Figure 5I with 5J; quantitation: Figure 6B and 6C), indicating that they are still associated with TSS. Thus, the TaxK7R and TaxK8R mutants do not change subcellular localization and their interactions with sc35 are not affected following UV irradiation.

Collectively, TaxM33, TaxK7R, or TaxK8R were all localized in the nucleus before and after UV irradiation (compare Figure 5C with 5D, Figure 5G with 5H, Figure 5I with 5J; quantitation Figure 6B) indicating that mutation of the NES, or of residues K280 or K284, independently inhibits DNA-damage dependent changes in Tax localization. However, these mutants displayed distinct differences in sub-nuclear localization. Mutation of the NES inhibited nuclear export but allowed Tax to dissociate from sc35. In contrast, mutation of ubiquitinated residues K280 or K284 prevented both nuclear export and dissociation from sc35 following DNA damage. These results suggest that ubiquitination of Tax at these residues allows it to be released from TSS, making it available for export from the nucleus in response to DNA damage.

## Discussion

Cellular transformation by HTLV-I is regulated, in part, by pleiotropic functions of the viral oncoprotein, Tax. The well recognized abilities of Tax to dysregulate cellular gene expression, cell cycle checkpoints, cell cycle progression, and DNA repair are partially due to its ability to interact with and alter the activity of cellular proteins [3]. Because understanding the regulation of interactions with cellular proteins that enable or disable Tax functions is essential to fully elucidate the mechanisms of Tax-mediated cellular transformation, it is the focus of intense interest. Tax exists both in the nucleus and in the cytoplasm of HTLV-I-infected or Tax-expressing cells and its nuclear and cytoplasmic functions are required for efficient cellular transformation [3]. Interactions between Tax and cellular proteins are not static and are affected by changes in the post-translational modification status of Tax, or as a consequence of exposure to genotoxic or cellular stress [11,13]. In these studies, ubiquitination of Tax or exposure to a number of DNA damaging or cellular stress-inducing agents resulted in cytoplasmic accumulation of the Tax protein.

In this study we examined the effects of DNA damage on the ubiquitination status of Tax and determined how DNA damage-induced changes in the post-translational modification status of Tax affected its subcellular localization and its interactions with host cell protein. In response to DNA damage, increased mono-ubiquitination of Tax was observed and mutation of known ubiquitinated residues, K280 and K284, prevented Tax ubiquitination, resulting in retention of Tax in nuclear foci together with the TSS marker protein, sc35. In addition, cytoplasmic accumulation of the ubiquitin-Tax fusion protein, regardless of whether ubiquitin was fused to the amino- or carboxyl-terminus, was inhibited by the CRM1-specific inhibitor, LMB [25,26]. LMB has been reported to inhibit stress-dependent nucleocytoplasmic shuttling of Tax, resulting in retention of the protein in nuclear speckles [11]. These results suggest that the subcellular localization of Tax and its interactions with cellular proteins following DNA damage are regulated, in part, by post-translational modifications, specifically ubiquitination of Tax at lysine residues 280 and 284.

Together with previously published data [11,13], our results suggest that the nuclear export of Tax, which occurs in response to DNA damage, is a multi-stage process. Following DNA damage, mono-ubiquitination of Tax at K280 or K284 facilitates its dissociation from cellular TSS proteins. This can be monitored by the reduced association of Tax with sc35 following DNA damage. CRM1 is then recruited to the Tax NES, which presumably becomes accessible following release from TSS proteins, resulting in formation of the Tax-CRM1 complex that mediates the nuclear export of Tax. Previous studies demonstrated that Tax exists in heterogeneous nuclear foci (TSS) and that the ability of Tax to interact with specific cellular proteins is controlled, in part, by post-translational modifications. Therefore, Tax molecules may respond differentially to DNA damage, depending on their modification status and/or interactions with host cell proteins. The heterogeneity of Tax interactions and post-translational modification status may explain the observation that not all Tax molecules are ubiquitinated following DNA damage, dissociate from sc35, or undergo nuclear export. Differences in Tax interactions may depend on the type of post-translational modification, the specific site of the modification, and/or the host-cell proteins that a Tax molecule is in complex with [12-14,18,28,29]. Additional studies will be required to clarify the variety of Tax interactions with host cell proteins, the impact of post-translational modifications on these interactions, and the collective contribution of these mechanisms to the subcellular localization of Tax under normal cellular conditions and in response to various stimuli.

It was recently reported that the nuclear export of Tax may be energy and carrier independent in unstressed cells [30]. In addition, Tax has been shown to interact with the calcium binding protein, calreticulin, at the nuclear membrane of astrocytes [31], suggesting a third potential mechanism of Tax export in which calreticulin directs nuclear export by binding to leucine-rich regions of the cargo protein and directing export in a LMB independent manner, [31-35]. Our current data, together with previously published work [11,22,30,31] present three potential mechanisms of Tax nuclear export which, on cursory glance, would appear to conflict with each other. However, all three mechanisms may be utilized by Tax under specific conditions, and additional factors may determine which mechanism is used by particular Tax molecules for nuclear export.

As previously discussed, Tax is a pleiotropic protein whose activities are regulated, in part, by formation of heterogeneous complexes with host cell proteins [3]. In addition, a number of studies suggest that the interaction of Tax with cellular proteins may be regulated by the post-translational modification status of Tax [12,14]. Therefore, with regard to nuclear export mechanisms, it is possible that the post-translational modification status of Tax and/or the cellular proteins with which it interacts may dictate the mechanism of nuclear export. Our studies, address nuclear export of Tax molecules that exist in TSS and specifically, those that interact with sc35, while the studies of Tsuji *et al*. [30] and Alefantis *et al. *[31] focus predominantly on diffuse nuclear Tax that is not in TSS, and Tax undergoing cellular secretion, respectively. In addition, our studies examine Tax molecules that have undergone changes in post-translational modification and subcellular localization following DNA damage. Therefore, it is possible that differences in nuclear export pathways reported by these three groups reflect the subpopulation of Tax being examined and illuminate three bona fide export processes. Additional studies will be required to fully elucidate the complexities of Tax nuclear export.

The effect of Tax localization on cellular responses to DNA damage remains to be characterized. However, the ubiquitination and cytoplasmic localization of Tax stimulates NF-κB activity through proteolytic and non-proteolytic mechanisms [7,13,16]. Cytoplasmic, ubiquitinated Tax in the form of UB-Tax fusion proteins, activate the NF-κB pathway by stimulating upstream kinases that interact with and target NF-κB inhibitor proteins for proteasomal degradation and by directly interacting with the NF-κB transcription factor p65/RelA in the cytoplasm and driving its nuclear import [7,13,16,36-38]. Although it remains to be examined, increased levels of cytoplasmic, ubiquitinated Tax that exist following DNA damage may induce NF-κB activity via a similar mechanism. Increased NF-κB activity or other enhanced cytoplasmic activities of Tax may dysregulate cellular responses to DNA damage. Additional studies will be required to fully understand the mechanism(s) by which ubiquitinated, cytoplasmic Tax dysregulates the cellular response to DNA damage.

## Conclusion

Previous studies have demonstrated that the oncogenic activities of HTLV-I Tax are regulated, in part, by its subcellular localization and interactions with numerous cellular proteins [11,13]. It was recently reported by us [11] and others [12,13] that the subcellular localization and interactions of Tax with cellular proteins are dynamic and can be influenced by various signals. The current study demonstrates that UV irradiation increases the mono-ubiquitination of Tax at lysine residues 280 or 284 and that these residues are essential for regulating the dissociation of Tax from cellular TSS proteins (sc35) and stimulating the nuclear export of Tax. Additional studies will be required to fully elucidate the biological consequences of Tax ubiquitination status, subcellular localization, and interactions with cellular proteins in response to DNA damage.

## Methods and materials

### Plasmids

pCMV-Tax and pCMV-M33 were previously described [24], pCMV-HAUB was provided by Dr. Wade Harper. pSG-Tax, pSG-TaxK1-10R, pSG-TaxK1-3R, pSG-TaxK4-10R, pSG-TaxK6-8R, pSG-TaxK6R, pSG-TaxK7R, pSG-TaxK8R, and pSG-TaxK78R expression vectors were provided by Claudine Pique [12]. The pSG-TaxUB expression vector was previously described [13] and was provided by Francoise Bex.

### Transfections

293 cells, which were maintained in Dulbecco's Modified Eagle Medium (DMEM) supplemented with 10% fetal bovine serum and grown at 37°C in 5% CO_2_, were transfected using Fugene6 (Roche, Indianapolis, IN), as described by the manufacturer.

### Antibodies

Anti-Tax antibodies Tab170 (AIDS Research and Reference Reagent Program, Germantown, MD) and 586 were previously described [11]. Anti-HA antibody was purchased from Bethyl Laboratories, Inc. (Montgomery, TX). Anti-sc35 antibody was purchased from BD-Pharmacia (San Diego, CA). Alexa-Fluor 594-conjugated goat anti-rabbit and Alexa-Fluor 488- conjugated goat anti-mouse secondary antibodies were purchased from Molecular Probes (Eugene, OR). Horseradish peroxidase conjugated secondary antibodies were purchased from Sigma-Aldrich (St. Louis, MO).

### UV irradiation

Media was removed from plates of transfected 293 cells and the cells were washed once with PBS. A Stratalinker (Stratagene, La Jolla, CA) was used to deliver 30 J/m^2 ^of UV-C irradiation. The original media was then replaced and the cells were allowed to recover for the specified length of time.

### Cellular Extracts

Whole cell extracts were prepared as previously described [39,40] with a few modifications. Briefly, 1 × 10^7 ^cells were resuspended in 20 μL of ice-cold buffer C (20 mM HEPES pH 7.9, 25% glycerol, 0.42 M NaCl, 1.5 mM MgCl_2_, 0.2 mM EDTA, 0.5 mM PMSF, 0.5 mM DTT) + 0.1% NP-40 with 10 mM N-ethylmaleimide (NEM) (Sigma-Aldrich, St. Louis, MO), incubated on ice for 10 minutes, and centrifuged for 10 minutes at 10,000 RPM at 4°C. The supernatant was diluted with 80 μL of ice-cold buffer D (20 mM HEPES pH 7.9, 20% glycerol, 50 mM KCl, 0.2 mM EDTA, 0.5 mM PMSF, 0.5 mM DTT) with 10 mM NEM (Sigma-Aldrich, St. Louis, MO) per 1 × 10^7 ^cells.

### Immunofluorescent Staining

Transfected 293 cells were washed once with PEM buffer (80 mM potassium PIPES pH 6.8, 5 mM EGTA pH 7.0, 2 mM MgCl_2_), and fixed by incubating in 5% formaldehyde diluted in PEM buffer for 30 minutes at 4°C. To remove excess formaldehyde, cells were washed three times in PEM buffer, and permeabilized by incubating in PEM buffer containing 0.5% Triton X-100 for 30 minutes at room temperature. Immunofluorescent staining was performed by incubating with primary antibody diluted in 2.5–5% BSA containing TBS + 0.1% Tween 20 (TBS-T) for a minimum of 3 hours at room temperature. Excess antibody was removed by washing cells three times in TBS-T. Cells were incubated in the dark with a fluorophore-conjugated secondary antibody diluted in TBS-T for 40 minutes at room temperature. Excess antibody was removed by washing the cover slips three times with TBS-T. The cells were counter-stained with DAPI (Sigma-Aldrich, St. Louis, MO) to visualize the nucleus, and mounted on slides using Slow-Fade Anti-fade mounting media (Molecular Probes, Eugene, OR). Cells were visualized on a Zeiss AxioPlan2 microscope using a CoolSnap HQ CCD camera and analyzed using MetaView, MetaMorph software.

### Immunoprecipitation

Whole cell extracts were prepared from 293 cells (~1.5 × 10^7^) that had been transfected with pSG-Tax, pSG-TaxK6R, or pSG-TaxK78R along with pCMV-HAUB. The extracts were incubated with anti-Tax (568) polyclonal antibody diluted in Incubation buffer (20 mM HEPES pH 7.9, 75 mM KCl, 2.5 mM MgCl_2_, 1 mM DTT, 0.1% NP-40, 0.5 mM PMSF, 1 μg/mL aprotinin, 1 μg/μL pepstatin, 2 μg/mL leupeptin, 1 mM sodium orthovanadate) overnight at 4°C on a rotator. 30 μL of a 50% protein G-bead slurry (Upstate, Lake Placid, NY) was added to the mixture and incubated for 90 minutes at 4°C. Beads were collected by centrifugation, washed 5 times with 500 μL of Incubation buffer, and resuspended in 100 μL SDS-PAGE sample buffer. Samples were resolved by SDS-PAGE (10%), transferred to a nitrocellulose membrane, and incubated with either anti-Tax (Tab170) or anti-HA antibodies. The membrane was then incubated with the appropriate HRP-conjugated secondary antibody.

## Competing interests

The author(s) declare that they have no competing interests.

## Authors' contributions

MLG and TD performed all experiments, participated in experimental design, and drafted the manuscript. SJM contributed to experimental design and preparation of the manuscript. All three authors have read and approved the final manuscript.
